# Functional Role of *Aspergillus carbonarius* *AcOTAbZIP* Gene, a bZIP Transcription Factor within the OTA Gene Cluster

**DOI:** 10.3390/toxins13020111

**Published:** 2021-02-02

**Authors:** Donato Gerin, Federica Garrapa, Ana-Rosa Ballester, Luis González-Candelas, Rita Milvia De Miccolis Angelini, Francesco Faretra, Stefania Pollastro

**Affiliations:** 1 Department of Soil, Plant and Food Sciences, University of Bari Aldo Moro, Via Giovanni Amendola, 165/A, 70126 Bari, Italy; donato.gerin@uniba.it (D.G.); f.garrapa1@studenti.uniba.it (F.G.); francesco.faretra@uniba.it (F.F.); stefania.pollastro@uniba.it (S.P.); 2 Food Biotechnology Department, Instituto de Agroquímica y Tecnología de Alimentos (IATA-CSIC), Calle Catedrático Agustín Escardino 7, 46980 Paterna, Valencia, Spain; ballesterar@iata.csic.es (A.-R.B.); lgonzalez@iata.csic.es (L.G.-C.); 3 SELGE Network of Public Research Laboratories, Via Amendola, 165/A, 70126 Bari, Italy

**Keywords:** *Aspergillus carbonarius*, OTA biosynthesis, bZIP transcription factor, gene deletion, gene expression, pathogenicity, secondary metabolism

## Abstract

*Aspergillus carbonarius* is the principal fungal species responsible for ochratoxin A (OTA) contamination of grapes and derived products in the main viticultural regions worldwide. In recent years, co-expressed genes representing a putative-OTA gene cluster were identified, and the deletion of a few of them allowed the partial elucidation of the biosynthetic pathway in the fungus. In the putative OTA-gene cluster is additionally present a bZIP transcription factor (*AcOTAbZIP*), and with this work, *A. carbonarius* Δ*AcOTAbZIP* strains were generated to study its functional role. According to phylogenetic analysis, the gene is conserved in the OTA-producing fungi. A *Saccharomyces cerevisiae* transcription factor binding motif (TFBM) homolog, associated with bZIP transcription factors was present in the *A. carbonarius* OTA-gene cluster no-coding regions. *AcOTAbZIP* deletion results in the loss of OTA and the intermediates OTB and OTβ. Additionally, in Δ*AcOTAbZIP* strains, a down-regulation of *AcOTApks*, *AcOTAnrps*, *AcOTAp450*, and *AcOTAhal* genes was observed compared to wild type (WT). These results provide evidence of the direct involvement of the *AcOTAbZIP* gene in the OTA biosynthetic pathway by regulating the involved genes. The loss of OTA biosynthesis ability does not affect fungal development as demonstrated by the comparison of Δ*AcOTAbZIP* strains and WT strains in terms of vegetative growth and asexual sporulation on three different media. Finally, no statistically significant differences in virulence were observed among Δ*AcOTAbZIP* strains and WT strains on artificially inoculated grape berries, demonstrating that OTA is not required by *A. carbonarius* for the pathogenicity process.

## 1. Introduction

Ochratoxin A (OTA) is a mycotoxin with nephrotoxic, carcinogenic, hepatotoxic, neurotoxic, immunosuppressive, and teratogenic effects, classified as a possible carcinogen in humans (group 2B) by the International Agency for Research in Cancer [1].

The fungi responsible for OTA contamination in agricultural products belong mainly to the genus *Aspergillus*, sections *Nigri* (e.g., *Aspergillus carbonarius* and *Aspergillus niger*), *Circumdati* (e.g., *Aspergillus steynii*, *Aspergillus westerdijkiae*), and *Flavi* (*A. albertensis* and *A. alliaceus*), and the genus *Penicillium* (e.g., *Penicillium nordicum* and *Penicillium verrucosum*) [2,3,4,5,6,7]. In the grapevine cultivated countries of the Mediterranean basin, *Aspergillus* species of the section *Nigri* occur more frequently and *A. carbonarius* is the largest producer of OTA in grape and grape-derived products [8,9].

Co-expressed genes, representing a putative-OTA gene cluster in *A. carbonarius* were identified by comparing the transcriptome of four OTA-producing strains grown under OTA-inducing and OTA-non inducing conditions. The cluster included a polyketide synthase (*AcOTApks*), a nonribosomal peptide synthase (*AcOTAnrps*), and halogenase (*AcOTAhal*) genes, proved to be directly involved in OTA biosynthesis [10,11,12], and additionally, a hypothetical protein recently annotated as cyclase [13], a cytochrome P450 monooxygenase (*AcOTAp450*) and a bZIP transcription factor (*AcOTAbZIP*) [14]. Recently, the same genes were identified by genomic diversity and RNA-Seq studies comparing *A. carbonarius* OTA producing and non-producing strains [15,16]. In addition, a consensus OTA biosynthetic pathway was identified in *A. ochraceus* fc-1 (recently re-classified as *A. westerdijkiae* [17]) by gene deletion approach demonstrating that the *AcOTApks*, *AcOTAnrps*, *AcOTAP450*, *AcOTAbZIP*, and *AcOTAhal* orthologue genes of *A. carbonarius* were directly involved in OTA biosynthesis [18]. 

Several transcription factors were found to regulate genes involved in the secondary metabolite biosynthesis. These include global transcriptional regulators as AreA (nitrogen regulation; [19,20]); PacC (pH regulation; [21]); CreA (carbon catabolite repressor; [22,23]); LaeA and VeA (light; [24]); metabolite-specific transcription factors such as AflR a Zn(II)2Cys6, regulating aflatoxins and sterigmatocistin biosynthetic genes [25]; Tri6 and Tri10 (both regulating the expression of trichotecene biosynthetic genes; [26]); and OTAR1 (a bZIP transcription factor involved in OTA biosynthesis in *A. westerdijkiae* fc-1 [18]).

bZIPs transcription factors are unique to eukaryotes and they are generally identified based on their bZIP domain, which includes a basic region (BR) and a leucine zipper (LZ). The BR is highly conserved, and it is characterized by an invariant N-x7-R/K region, while the LZ is composed of several repeats of leucine or other bulky hydrophobic amino acids (Ile, Val, Phe, or Met), and it is arranged exactly nine amino acid residues toward the C-terminus of the BR [27]. bZIP monomers are long α-helices that bind specific DNA sequences through the BR and interact through the LZ that mediates the dimerization to form a superimposed coiled-coil structure [28]. This structure, therefore, affects binding characteristics, expression diversity, and gene regulation of the target genes [27,28].

In this study, we deleted the *A. carbonarius AcOTAbZIP* gene, a bZIP transcription factor included in the putative OTA gene cluster and conserved in OTA-producing fungi. Three deletion mutants were selected and compared with the wild type (WT) for OTA production, vegetative growth, asexual sporulation, and colonization of grape berries by artificial inoculation. Chemical analyses of the OTA-intermediates and gene expression studies were also performed to assess the *AcOTAbZIP* role in the *A. carbonarius* OTA-biosynthetic pathway.

## 2. Results

### 2.1. Characterization of AcOTAbZIP Gene

**Figure 1:**
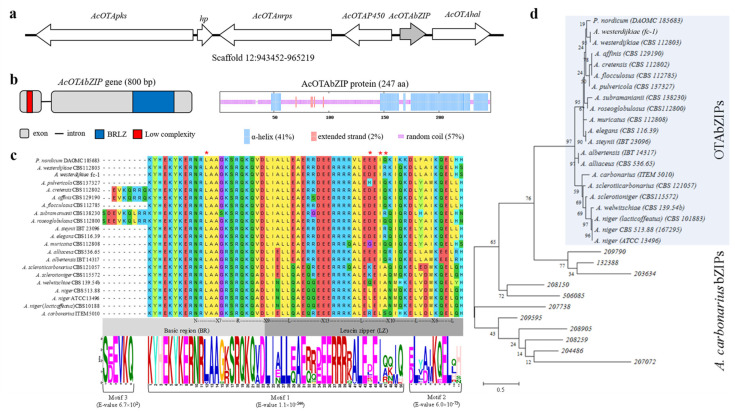
Characterization of *AcOTAbZIP* gene. (**a**) location of *AcOTAbZIP* within the *A. carbonarius*-OTA gene cluster containing also the *AcOTApks*, a hypothetical protein (*hp*, recently annotated as cyclase [13]) coding gene, the *AcOTAnrps*, the *AcOTAP450*, and the *AcOTAhal* genes; (**b**) in silico analysis of *AcOTAbZIP* gene and related proteins; (**c**) alignment of the BR-LZ domain predicted by SMART into each OTAbZIP protein and relative motifs predicted by MEME; (**d**) phylogenetic analysis by using Maximum Likelihood (ML) method and JTT matrix-based model. In c, red asterisks indicate the amino acids unique to *Aspergillus carbonarius*. In d, the percentage of trees in which the associated taxa clustered together is shown next to the branches; the tree is drawn to scale, with branch lengths measured in the number of substitutions per site.

The *A. carbonarius AcOTAbZIP* gene is located in the scaffold 12 of *A. carbonarius* genome; it is 800 bp in length and encodes a protein of 247 aa (Figure 1a,b). Its orthologues were found in 20 *Aspergillus* and *Penicillium* species, and they were located in a putative OTA-biosynthetic gene cluster (Table S1).

Based on the fungal BRLZ domain alignment and the motif prediction of BRLZ domains, it was possible to identify the invariant N-X7-R region typical of the BR domain, the R-X9-L region that allows distinguishing the BR domain and LZ domain and, at least, four leucine residues in the LZ domain common to all examined fungal species. Additionally, the BRLZ domain of *A. carbonarius* showed four unique amino acid substitutions in the positions 12 (V/L), 44 (R/E,D,H,G,K,Q,L), 46 (L/I), and 47 (S/Q,R,A), respectively in the motif 1 identified by MEME analysis (Figure 1c).

According to the BRLZ-phylogenetic analysis, the tree with the highest log likelihood (−2212.83) is shown in Figure 1d. ML analysis showed that the other 11 *A. carbonarius* bZIP transcription factors annotated in the genome and carrying the BRLZ domain were clustered separately to the OTAbZIP transcription factors of *Aspergillus* spp. and *Penicillium nordicum*. According to the ML tree, the subsequent OTAbZIPs were grouped in: (i) *A. carbonarius* ITEM 5010, (ii) *A. niger* strains CBS 101883, ATCC 13496 and CBS 513.88, *A. sclerotiicarbonarius* CBS 121057, *A. sclerotioniger* CBS 115572 and *A. welwitschiae* CBS 13954b (section *Nigri*), (iii) *A. albertensis* IBT 14317 and *A. alliaceus* CBS 536.65 (section *Flavi*), and (iv) *A. affinis* CBS 129190, *A. cretensis* CBS 112802, *A. elegans* CBS 116.39, *A. flocculosus* CBS 112785, *A. muricatus* CBS 112808, *A. pulvericola* CBS 137327, *A. roseoglobulosus* CBS 112800, *A. steynii* IBT 23096, *A. subramanianii* CBS 138230, *A. ochraceus* fc-1 and *A. westerdijkiae* CBS 112803 (section *Circumdati*), and *P. nordicum* DAOMC 185683 (Figure 1d, Table S2).

The most representative TFBM found by MEME in all fungal species was 15 bases in length (RATGACGTGTARANV) and it occurred in 129 sites into the provided sequences (e-value = 3.1 × 10^−160^) (Table S3). Additionally, according to TOMTOM analysis, the predicted TFBM showed homology (*p*-value ≤ 0.01) with TFBM of *Saccharomyces cerevisae* related to bZIP transcription factors and other classes, such as tryptophan cluster factors, basic helix-loop-helix factors (bHLH), TALE-type homeodomain factors, and APSES-type DNA-binding domain (Table S4).

### 2.2. Generation of A. carbonarius Deletion Mutants

**Figure 2:**
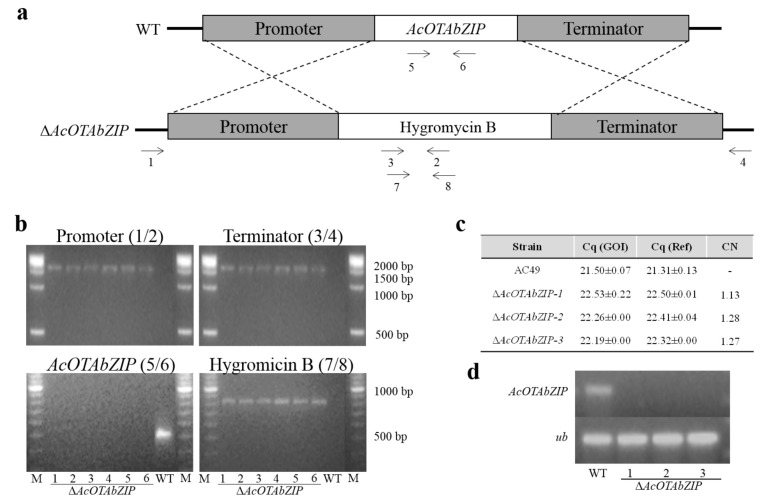
Generation of Δ*AcOTAbZIP* strains. (**a**) Strategy of gene replacement; primer pairs AcOTAbZIP_1F (1)/HPH1F (2), HPHPRO4 (3)/AcOTAbZIP_2R (4), AcOTAbZIP_3F (5)/AcOTAbZIP_4R (6) and HMBR1 (7)/HMBF1 (8) were used for the amplification of promoter, terminator, AcOTAbZIP and Hygromycin B in the *AcOTAbZIP* locus, respectively. (**b**) PCR pattern including the promoter, terminator, AcOTAbZIP, and Hygromycin B amplification products; (**c**) copy number analysis by qPCR; GOI is AcOTAbZip, Ref is calmodulin and CN indicates copy number. (**d**) RT-PCR analysis of the *AcOTAbZIP* gene and the reference gene ubiquitin (*ub*).

To investigate the role of *AcOTAbZIP* in OTA biosynthesis, the gene was deleted in the *A. carbonarius* AC49 strain by replacement with the hygromycin resistance cassette (Figure 2a). After co-cultivation of *A. carbonarius* (1.5 × 10^4^ conidia plate^−1^) with *A. tumefaciens* AGL-1 carrying the pRFHU2-AcOTAbZIP plasmid an average of 17 *A. carbonarius* HygB-resistant colonies per plate were obtained (efficiency: 0.11%). Monosporic isolates were obtained after three subcultures on PDA containing 100 µg mL^−1^ hygromycin B (InvivoGen, San Diego, CA, USA), and the PCR pattern corresponding to homologous integration of T-DNA in the target site was assessed (Figure 2b).

Three selected Δ*AcOTAbZIP* mutants were also assayed for evaluating the number of T-DNA copies integrated into the genome by qPCR using the WT parental strain as control (Figure 2c). The three deletants contained one single event of integration. Finally, the three Δ*AcOTAbZIP* mutants were subjected to RT-qPCR analysis to demonstrate that the *AcOTAbZIP* gene was not functionally present in the genome of the mutants (Figure 2d). The three Δ*AcOTAbZIP* mutants were then used for the subsequent analyses.

### 2.3. Phenotypic Characterization

**Figure 3:**
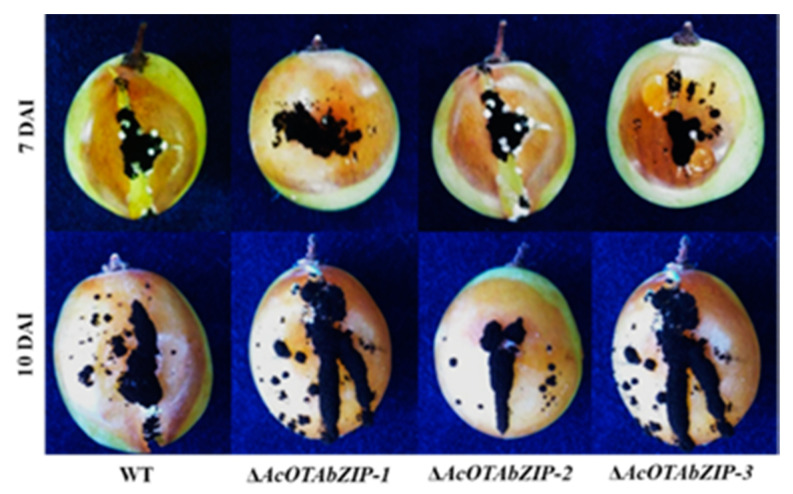
Colonization of wilt type (WT) e deletant (Δ*AcOTAbZIP*) *A. carbonarius* strains of grape berries of cultivar Italia.

No statistical differences regarding in vitro fungal growth and sporulation, and virulence on artificially inoculated grape berries were observed for the three Δ*AcOTAbZIP* mutants compared to the WT strain (Table 1, Figure 3). Under in vitro conditions, 7 DAI at 25 °C, the daily growth rate was 4.2–4.3 mm day^−1^ on MM, 7.5–7.6 mm day^−1^ on PDA, and 6.4 mm day^−1^ on MEA for Δ*AcOTAbZIP* mutants and the WT strain. Seven DAI at 25 °C all strains produced up to 10.6 × 10^4^ conidia/mm^2^ on MM, 0.4 × 10^4^ conidia/mm^2^ on PDA and 0.5 × 10^4^ conidia/mm^2^ on MEA. Daily growth rate of the rotted area on grape berries was 2.9–3.0 mm day^−1^ on cv Italia and 2.2–2.3 mm day^−1^ on the cv Red Globe (Table 1).

### 2.4. Chemical Analysis of OTA and Its Intermediates and Gene Expression Studies

**Figure 4:**
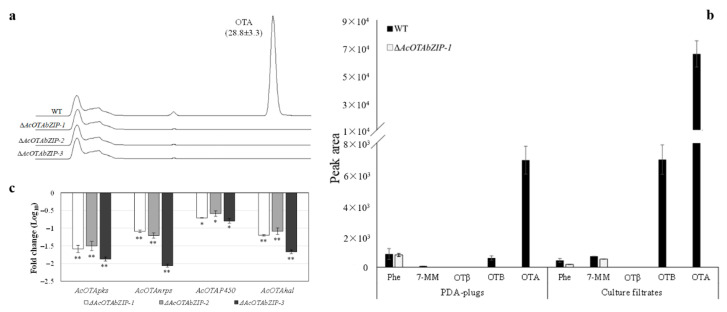
Chemical analysis of OTA and its related compounds and gene expression study in WT and Δ*AcOTAbZIP* strains. (**a**) Preliminary HPLC analysis of OTA and its intermediates [phenyalanine (Phe), 7-methylmellein (7-MM), ochratoxin β (OTβ), ochratoxin B (OTB)]; (**b**) HPLC-HRMS analysis of OTA and its intermediate metabolites. Data are the average value ± standard error; (**c**) Gene expression analysis. The relative expression value of the three Δ*AcOTAbZIP* strains was compared with that of the WT (fold change). Data are the average value ± standard error. Significant differences in the relative expression of biosynthetic genes between Δ*AcOTAbZIP* strains and WT were assessed at *p* ≤ 0.05 (*) and *p* ≤ 0.01 (**) by using Tukey’s test.

After 7 days of growth on PDA, the Δ*AcOTAbZIP* strains did not produce OTA compared to WT, which produced 28.8 ± 3.3 ng µL^−1^ (Figure 4a).

Methanol extracts of PDA-plugs collected from WT and Δ*AcOTAbZIP* cultures were also analyzed for the presence of Phe, 7-MM, OTβ, OTB, and OTA by HPLC-HRMS (Figure 4b). The Phe level was similar in the WT and Δ*AcOTAbZIP* strains in PDA plugs (peak area: 854 and 797, respectively); however, the amount of Phe detected on cultural filtrates was about 2-fold higher in the WT compared to Δ*AcOTAbZIP* strains. Low levels of 7-MM were only detected in the PDA-plugs of the WT strain (peak area: 81), and 7-MM was not detected in those of the Δ*AcOTAbZIP* strain. In the culture filtrates, 7-MM was detected in both WT and Δ*AcOTAbZIP* strains. Levels of this compound were higher for the WT compared to the deletant strain (peak area: 713 and 527, respectively). No OTβ was detected in either PDA-plugs or culture filtrates of WT and Δ*AcOTAbZIP* strains. Finally, levels of OTB and OTA were only detected in both PDA-plugs and culture filtrates of the WT, with a peak area of OTB in PDA-plugs and culture filtrate of 599.7 and 6934 respectively, and OTA of 6897.7 and 65,359 (Figure 4b).

To verify the role of *AcOTAbZIP* as the regulator of expression of the OTA biosynthetic genes, a gene expression study was carried out by RT-qPCR. The results showed that after 4 days of growth under OTA inducing conditions, the expression of the OTA biosynthetic genes (*AcOTApks*, *AcOTAnrps*, *AcOTAP450*, and *AcOTAhal*) was significantly down-regulated in the Δ*AcOTAbZIP* strains (*p* ≤ 0.05) compared to WT (Figure 4c).

## 3. Discussion

According to the current knowledge, the *A. carbonarius*-putative OTA gene cluster includes five biosynthetic genes: (i) the *AcOTApks* gene encoding the polyketide synthase (PKS) involved in the synthesis of the dihydrocoumarin moiety [11]; (ii) a hypothetical protein, recently annotated as cyclase putatively involved in the polyketide cyclization during the initial step; [13] (iii) the *AcOTAP450* gene coding for the cytochrome P450 monooxygenase putatively involved in the dihydrocoumarin C7-oxidation; (iv) the *AcOTAnrps* gene encoding the non-ribosomal peptide synthase (NRPS) responsible of the peptide bond between dihydrocoumarin with the L-phenylalanine, originating OTB [10]; and (v) the *AcOTAhal* gene coding for the halogenase (chloroperoxidase) that provides the addition of a chlorine atom to obtain OTA [12]. A fifth highly conserved gene, a basic leucine zipper transcription factor *AcOTAbZIP*, has also been described to be part of the biosynthetic gene cluster; however, the role of this transcription factor in the OTA biosynthesis of *A. carbonarius* is still unclear. The present study aimed to functionally characterize this transcription factor by using the ATMT based gene deletion approach and to determine the involvement of this transcription factor in the OTA biosynthesis in *A. carbonarius*. 

The recent availability of fungal genomes allowed the identification of *AcOTAbZIP*-orthologue genes being part of putative gene clusters in other 20 OTA-producing strains. Recently, the putative OTA gene cluster was identified in *A. westerdijkiae* fc-1 and the deletion of the *AcOTAbZIP*-orthologue gene (*OtaR1*) blocked the OTA biosynthesis [18]. These authors also proposed that *OtaR1* is probably a pathway-specific regulator that controls OTA production by regulating the biosynthetic genes in *A. westerdijkiae* fc-1.

In our study, the BR-LZ domain of OTAbZIP proteins of *Aspergillus* spp. and *P. nordicum* clustered together, and they were separated from the BR-LZ domains of other *A. carbonarius*-bZIP transcription factors, indicating their conserved specialization in the OTA biosynthesis. The prediction of TFBMs in the upstream, downstream, and intergenic regions of the *Aspergillus* spp. and *P. nordicum* putative OTA gene cluster allowed the identification of a TFBM of 15 bp including a conserved “TGACGTGTA” sequence. This result is in agreement with previous work that showed the identification of this conserved feature in the OTA gene cluster of five OTA-producing species (*A. carbonarius*, *A. niger*, *Aspergillus steynii*, and *Aspergillus westerdijkiae* and *P. nordicum*) [29].

Although several mycotoxin biosynthetic pathways have been elucidated, relatively little is known about the molecular mechanisms of OTA biosynthesis. The hypothesis is that the biosynthesis of OTA follows the subsequent pathway: backbone polyketide (possibly 7-MM)→OTβ→OTB→OTA [12,30]. Recent advances on the understanding of the OTA biosynthetic pathway were performed by analyzing OTA and its intermediates in both WT and deletion mutants of *AcOTApks*, *AcOTAnrps*, and *AcOTAhal* biosynthetic genes [10,11,12]. In our study, the deletion of *the* *AcOTAbZIP* gene blocks the OTA biosynthesis. According to the HPLC-HRMS analysis, Δ*AcOTAbZIP* strains were unable to synthesize OTB, the precursor of OTA. Additionally, both WT and Δ*AcOTAbZIP* strains did not produce OTβ probably due to its rapid conversion into OTB in the case of WT and likely due to the deletion of the gene in the Δ*AcOTAbZIP* strains. Finally, 7-MM, proposed as a possible backbone polyketide of the OTA biosynthetic pathway [31], was produced by both the WT and the Δ*AcOTAbZIP* strains in culture filtrates and only by WT in PDA-plugs. Probably, contrary to what happens with the other OTA intermediates (OTβ and OTB), 7-MM represents the backbone structure for the biosynthesis of different polyketides, including OTA. 

**Figure 5:**
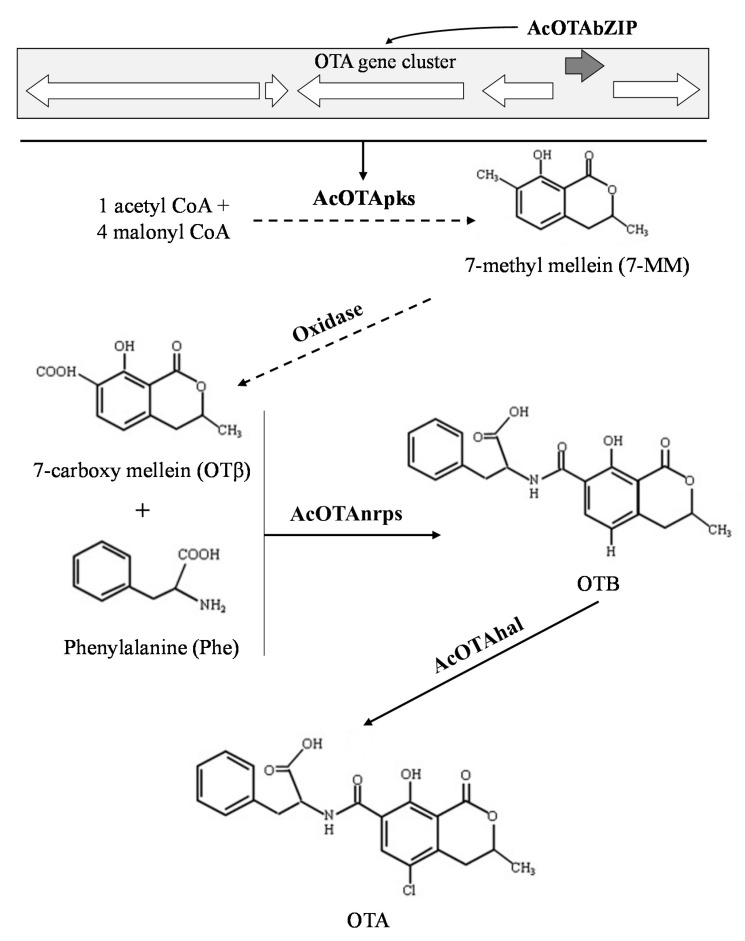
Schematic description of the OTA biosynthetic pathway according to [12] and herein obtained results, resuming the functional role of AcOTAbZIP, AcOTApks, AcOTAnrps, and AcOTAhal. Dashed arrows represent the hypothesized enzymatic steps.

The gene expression analysis showed that all analyzed genes (*AcOTApks*, *AcOTAnrps*, *AcOTAp450*, and *AcOTAhal*) were down-regulated in Δ*AcOTAbZIP* compared to WT strains. Similar results were obtained by the deletion of the *AcOTAbZIP* orthologue gene *OtaR1* in *A. westerdijkiae* fc-1 in which this gene regulates the expression of the OTA biosynthetic genes. Castellá et al., [16] have also indicated that the down-regulation of the *AcOTAbZIP* may explain the lack of OTA production in the three non-ochratoxigenic strains analyzed, indicating that this transcription factor is the major regulator of the OTA biosynthesis in *A. carbonarius* (Figure 5).

It is well known that in filamentous fungi, different genes can be involved in both fungal development and secondary metabolism [32]. For example, in *A. carbonarius* the deletion of the *alb1* gene, the pks involved in the 1,8-dihydroxynaphtalene melanin biosynthesis, affects conidia pigmentation, conidia, and sclerotia production, and the production of OTA and its partitioning into the fungal structures [33]. It has been reported that the deletion of *AcpacC* gene, a pH-responsive transcription factor, affects vegetative growth, conidia production, and germination, and OTA production [34]. In the present study, the deletion of the *AcOTAbZIP* gene did not affect the vegetative growth and conidia production of *A. carbonarius* growing on different media (MEA, MM, and PDA) compared to WT. The involvement of mycotoxins in the infection process is still under study in the producer fungi. For example, gliotoxin and trichothecenes are recognized as important virulence factors of *A. fumigatus* and *Fusarium graminearum*, respectively [35,36]. For patulin, no difference was observed in terms of pathogenicity between patulin producing (PEXP and PEX1) and non-producing (PEX2) *P. expansum* wild strains. Additionally, in the same fungus, the deletion of *patK*, *patL*, and *patN*, genes directly involved in patulin biosynthesis, resulted in the lack of ability to produce the mycotoxin but not in differences in growth rate, sporulation, and pathogenicity on apple fruits with respect to WT [37]. In our study, both WT and Δ*AcOTAbZIP* strains were inoculated on berries of two table grape cultivars (Italia and Red Globe) to understand the role of OTA as a virulence factor. No differences were observed between WT and Δ*AcOTAbZIP* strains, suggesting that under the tested conditions, OTA is not involved in the plant tissue colonization of *A. carbonarius* on grape berries.

In conclusion, the present study showed the functional role of the *AcOTAbZIP* gene on the OTA biosynthesis. This gene is conserved in the species carrying a putative-OTA gene cluster and is directly involved in the OTA pathway by regulating the expression of the four biosynthetic genes *AcOTApks, AcOTAp450*, *AcOTAnrps*, *AcOTAhal*. No differences in terms of fitness were observed between WT and Δ*AcOTAbZIP* strains suggesting that OTA is not involved in the virulence of *A. carbonarius* on grapes.

## 4. Material and Methods

### 4.1. Strains and Media

The OTA-producing AC49 strain of *A. carbonarius* was used as WT (Gerin et al., 2016; 2018), to generate Δ*AcOTAbZIP* deletion mutants. All strains were routinely grown on potato dextrose agar (PDA; infusion from 200 g peeled and sliced potatoes kept at 60 °C for 1 h, 20 g dextrose, adjusted at pH 6.5, 20 g agar Oxoid no. 3, per liter). Minimal medium [MM; 10 mL solution A (10 g KH_2_PO_4_, per 100 mL of water), 10 mL solution B (20 g NaNO_3_, 5 g KCl, 5 g MgSO_4_·7H_2_O, 0.1 g FeSO_4_, per 100 mL of water), 1 mL of micro-nutritive solution [38], 20 g glucose, 20 g agar Oxoid no. 3, per liter], malt extract agar (MEA: 20 g malt extract and 20 g agar Oxoid no. 3, per liter) and PDA were used in phenotypic characterization and to evaluate OTA production.

### 4.2. Identification and Characterization of AcOTAbZIP Gene

The features of the *AcOTAbZIP* gene were analyzed in the Doe Joint Genome Institute portal (https://mycocosm.jgi.doe.gov/cgi-bin/dispGeneModel?db=Aspca3&id=7821). The whole protein sequence was submitted to NPS@ using the “Secondary structure consensus prediction” tool [39] to predict α-helices, random coil, and other protein features.

The AcOTAbZIP transcription factor (ID: 7821) of *A. carbonarius* was used for the BLASTp analysis to identify the orthologue genes in other *Aspergillus* and *Penicillium* species (https://genome.jgi.doe.gov). For each fungal species, the identified gene was considered an OTAbZIP transcription factor if it was clustered with the orthologue genes of *A. carbonarius AcOTApks*, *AcOTAnrps*, *AcOTAP450*, and *AcOTAhal*. All OTAbZIP proteins of different fungal species and also all bZIP proteins present in the *A. carbonarius* genome were downloaded (https://genome.jgi.doe.gov). The nucleotide sequence of OTAbZIP was firstly identified into the *A. westerdijkiae* fc-1 assembled genome (www.ncbi.nlm.nih.gov/assembly/GCA_004849945.1), by BLASTn using the *A. westerdijkiae* CBS 112803 OTAbZIP as the query sequence, because the proteome of the target fungus is still lacking. Then protein sequence was obtained by using the ExPaSy translation tool (http://expasy.org/tools/dna.html). For each bZIP sequence, the BRLZ domain was obtained by using SMART [40] and used for performing phylogenetic analysis with the Maximum Likelihood method (ML) and JTT matrix-based model in MEGAX software [41].

To better identify the conserved regions (N-x7-R/K, into BR and leucine repeats into LZ) into BRLZ, domains motifs were predicted by using Multiple EM for Motif Elicitation (MEME) tool in the Motif-based sequence analysis tools (MEME Suite 5.1.0; [42]). Additionally, the MEME tool was used to examine the presence of the putative-Transcription Factor Binding Motifs (TFBMs) into the entire nucleotide sequences of upstream, downstream, and intergenic regions of each putative OTA-gene cluster in the listed OTA producing fungi (Table S1). For *A. carbonarius*, untranslated (UTR) regions of each gene of the cluster were also included, because it was recently reported that the control of gene expression can be UTR-dependent [43]. The most representative motif was then used in the Motif Comparison Tool (Tomtom, MEME Suite 5.1.0) to analyze its similarity with TFBMs present in the motif database of JASPAR CORE (2018) fungi with a cut-off *p*-value of 0.01.

### 4.3. Deletion of AcOTAbZIP Gene in A. carbonarius

All primer pairs were designed with the Primer3 software [44]. The amplification of the promoter and the terminator regions (~1.5 kb) from *A. carbonarius* AC49 genomic DNA was performed using Top-Taq DNA polymerase (Bioron GmbH, Ludwigshafen, Germany), according to the manufacturer’s instructions, and using the primer pairs AcOTAbZIP_O1/AcOTAbZIP_O2 and AcOTAbZIP_A3/AcOTAbZIP_A4 for the promoter and terminator regions of the *AcOTAbZIP* gene, respectively (Table 2). PCR conditions were 94 °C for 3 min, 35 cycles of 94 °C for 15 s, 58 °C for 20 s, and 72 °C for 2 min, and a final stage at 72 °C for 10 min. The plasmid pRFHU2-AcOTAbZIP was obtained according to Frandsen et al. [45] by incubating the promoter, terminator, and *PacI*/*Nt.BbvCI*-digested pRFHU2 (ratio 30:30:120 ng) and 1 µL of the Uracil-Specific Excision Reagent (USER) enzyme (New England Biolabs, Ipswich, MA, USA) at 37 °C for 20 min followed by 25 °C for 20 min.

Aliquots (10 µL) of the mixture were used for the transformation of chemically competent cells of *E. coli* DH5α [45]. After 18 h of incubation at 37 °C on Luria-Bertani (LB) agar medium (bacto tryptone 10 g, yeast extract 5 g, NaCl 5 g, agar 14 g, per liter) supplemented with 25 µg mL^−1^ of kanamycin (Invitrogen, Carlsbad, CA, USA), resistant colonies were first screened by PCR using the primer pairs RF-5/RF-2 and RF-1/RF-6 (Table 2) and the fusion was confirmed by using the primers RF-2/AcOTAbZIP_O1 and RF-1/AcOTAbZIP_A4.

The plasmid pRFHU2-AcOTAbZIP was then introduced in electrocompetent cells of *A. tumefaciens* AGL-1. *A. carbonarius* AC49 transformants were obtained and screened according to the *Agrobacterium tumefaciens* mediated transformation (ATMT)-procedure described by Gerin et al. [33]. In particular, for all transformants the integration of the T-DNA in the target region was verified by PCR by using the subsequent primer pairs: (a) 1F/HPH1F for the promoter; (b) 2R/HPHPRO4 for the terminator; (c) 3F/4R for the deletion of the gene of interest (GOI), and (d) primers HMBF1/HMBR1 for replacement of GOI with HygB. The determination of the number of T-DNA integrations in the genome of the transformants was assessed by qPCR (primers 3F/4R) using the calmodulin gene (cal; ID: 205510 primers cal_CN_F/cal_CN_R) as a reference (Table 2), as described by Gerin et al. [33].

### 4.4. Phenotypic Characterization and In Vivo Assay

Three *A. carbonarius AcOTAbZIP* (Δ*AcOTAbZIP-1*, Δ*AcOTAbZIP-2*, and Δ*AcOTAbZIP-3*) deletion mutants were selected and compared with the WT strain for colony growth and production of conidia on three different media (PDA MM and MEA). Mycelial plugs of 4 mm in diameter from the edges of actively growing colonies were used to inoculate three replicated Petri dishes that were kept at 25 ± 1 °C in the darkness. The orthogonal diameters of developing colonies were measured at 2, 5, and 7 days after inoculation (DAI). Additionally, the production of conidia was determined in three agar plugs (4 mm diameter) with mycelium and conidia collected from the inner, middle, and outer positions of 7-day-old growing colonies [24].

For each strain, three replicated groups of five ripe table-grape berries cvs Italia and Red Globe collected from two bunches each were surface-sterilized with 2% sodium hypochlorite for 1 min, rinsed three times with sterile distilled water, and airdried. Conidia were collected by scraping in sterile water containing 0.01% tween 20 the surface of 7-day-old colonies grown on PDA, and suspensions were adjusted to 10^6^ conidia mL^−1^. Aliquots (10 µL) of the conidial suspensions were singly placed on the berry skin, which was then wounded with a needle (3-mm-deep) under the drop. Berries were kept under 100% relative humidity. After 7 and 10 DAI at 25 ± 1 °C in darkness, the orthogonal diameters of the developing lesion were measured. Ten replicated berries inoculated with sterile water were used as control.

In both, in vitro and in vivo assays, the growth rate of colonies or rotted areas (mm day^−1^) was obtained from the average of the ratios between the diameters (mm) and the number of days of incubation (2, 5, and 7 days for in vitro colony growth assay and 7 and 10 days for in vivo pathogenicity assay, respectively).

### 4.5. Analysis of OTA and Its Intermediates

The preliminary analysis of OTA production by *A. carbonarius* WT and Δ*AcOTAbZIP* strains was performed on methanol extracts from agar plugs collected from 7 DAI colonies grown on PDA. Briefly, three 6-mm plugs of PDA (collected from the inner, middle, and outer part of the colony) were vortexed for 2 min in 500 µL of methanol, incubated at room temperature for 1 h, filtered on a 0.22 µm filter, and stored at −20 °C until the HPLC analysis which was performed as described by Gerin et al. [33]. No differences in terms of OTA production among the three selected Δ*AcOTAbZIP* strains were observed and then only the Δ*AcOTAbZIP-1* and the WT strain were used for the subsequent analysis of OTA and its possible intermediates. The analyses were performed on agar plugs from 7-DAI colonies grown on PDA as well as from culture filtrates from MM static cultures at 6 DAI [14]. Three technical replicates were performed. OTA and its intermediates (phenylalanine (Phe), 7-methylmellein (7-MM), ochratoxin β (OTβ), ochratoxin B (OTB) [10,12]) were quantified with a TripleTOF 5600 (AB SCIEX, Framingham, MA, USA) LC/MS/MS System with electrospray ionization operated in positive mode. The column was a Kinetix XB-C18 column (100 mm by 2.1 mm, 1.7 µm particles, 100 Å; Phenomenex Inc., Torrance, CA, USA). The mobile phase was a multistep gradient of water (eluent A) and methanol (eluent B), both containing 0.5% acetic acid and 1 mM ammonium acetate. Gradient elution was performed by changing the mobile phase composition as follows. After 5 min at 20% eluent B, the proportion was set at 40% and then linearly increased to 63% in 30 min and kept constant for 5 min. The column was re-equilibrated with 20% eluent B for 10 min before the successive injection. The data acquisition used was in positive mode, over a mass range of 80–1000 *m*/*z*. Automated calibration was performed using an external calibrant delivery system (CDS) which infuses calibration solution before sample introduction. The MS analysis was performed with the following parameters: 5500 V ion spray voltage (ISVF); 30 V collision energy (CE); 350 °C temperature with 30 psi curtain gas (CU); 50 psi for both ion source gas 1 (GC1) and ion source gas 2 (GS2). Data were evaluated using the PeakViewTM software.

### 4.6. Gene Expression Studies by RT-qPCR

Total RNA was extracted by 4-day-old cultures of Δ*AcOTAbZIP*-1/3 and WT strains grown in liquid MM in darkness at 25 ± 1 °C (OTA inducing conditions, [14]) using the RNeasy Plant Mini Kit (Qiagen, Milan, Italy) according to the manufacturer’s instructions. First-strand cDNA was synthesized from 1 μg of RNA using M-MLV reverse transcriptase (Life Technologies, Milan, Italy) and random primers in a volume of 20 μL, according to the manufacturer’s instructions. The expression of genes included in the putative OTA gene cluster (*AcOTApks*, *AcOTAnrps*, *AcOTAP450*, and *AcOTAhal*) was assessed by using a real-Time PCR Detection System CFX96TM (Bio-Rad Laboratories, Hercules CA, USA) in a volume of 25 μL containing 12.5 μL of iQ SYBR Green SuperMix (Bio-Rad Laboratories), 0.5 μM of each primer and 1 μL of the reverse transcription reaction. All primer pairs were designed with the Primer3 software, and where possible, the forward ones were designed on the exon-intron junction sites to avoid amplification of possible contaminant genomic DNA (Table S1). The conditions for amplification were as follows: 3 min denaturation at 95 °C followed by 35 cycles of 95 °C for 10 s and 60 °C for 45 s. The gene encoding ubiquitin (*ub*; ID:393986) was used as a reference gene. Relative gene expression was calculated using CFX Manager Software (Bio-Rad Laboratories) and the 2^−ΔΔCT^ method [46]. All samples were analyzed in triplicate. For all analyzed genes, the ratio of the gene expression value (fold change) between each deletion mutants and the WT strain was calculated.

## Figures and Tables

**Table 1:** Phenotypic characterization of Δ*AcOTAbZIP* compared to WT.

Strain	In Vitro Assay						Assay on Grape Berries	
	Growth Rate (mm day^−1^)			Conidia [(No. × 10^4^)/mm^2^]			Growth Rate (mm/day)	
	MM	PDA	MEA	MM	PDA	MEA	Italia	Red Globe
WT	4.2 ± 0.3 a	7.6 ± 0.7 a	6.4 ± 0.3 a	8.2 ± 0.2 a	0.4 ± 0.4 a	0.5 ± 0.1 a	3.0 ± 0.1 a	2.3 ± 0.1 a
Δ*AcOTAbZIP-1*	4.3 ± 0.3 a	7.5 ± 0.7 a	6.4 ± 0.3 a	10.6 ± 1.0 a	0.4 ± 0.3 a	0.5 ± 0.1 a	2.9 ± 0.1 a	2.2 ± 0.1 a
Δ*AcOTAbZIP-2*	4.3 ± 0.3 a	7.5 ± 0.7 a	6.4 ± 0.4 a	7.5 ± 1.1 a	0.4 ± 0.1 a	0.5 ± 0.2 a	3.0 ± 0.1 a	2.3 ± 0.1 a
Δ*AcOTAbZIP-3*	4.3 ± 0.3 a	7.5 ± 0.7 a	6.4 ± 0.4 a	7.5 ± 1.1 a	0.4 ± 0.2 a	0.5 ± 0.1 a	3.0 ± 0.2 a	2.2 ± 0.1 a

Data represent mean values ± standard error. The growth rate (mm day^−1^) was obtained from the mean values of the ratios between the growth (mm) and the number of days (2, 5, and 7 days for in vitro assay and 7 and 10 days for in vivo assay, respectively). For each column, values followed by the same letter means did not differ statistically at *p* ≤ 0.05 according to Tukey’s test. MM, PDA, and MEA: minimal medium, potato dextrose agar, and malt extract agar media, respectively.

**Table 2:** Primers used for the generation, validation, and gene expression analysis of *Aspergillus carbonarius* Δ*AcOTAbZIP* strains.

Target Region	Primer Name	Primer Sequence (5′-3′)
Promoter and terminator amplification in *A. carbonarius* (AC49)		
*AcOTAbZIP* promoter	AcOTAbZIP_O1	**GGTCTTAAU**TGTTGAAGGTGCGGTTCTTG
	AcOTAbZIP_O2	**GGCATTAAU**CATGAGCATTGACACGAGCC
*AcOTAbZIP* terminator	AcOTAbZIP_A3	**GGACTTAAU**TGAGCGCATGTCTAGCAAAC
	AcOTAbZIP_A4	**GGGTTTAAU**TCGGCCGTGAAGCAGTTATA
Screening in *E. coli* (DH5α)		
pRFHU2-*AcOTAbZIP* plasmid	RF-2	TCTCCTTGCATGCACCATTCCTTG
	RF-5	GTTTGCAGGGCCATAGAC
	RF-1	AAATTTTGTGCTCACCGCCTGGAC
	RF-6	ACGCCAGGGTTTTCCCAGTC
Screening in *A. carbonarius* (AC49 and Δ*AcOTAbZIP* strains)		
*AcOTAbZIP* promoter	AcOTAbZIP_1F	AGGCGTTATAGGACCAGTCG
	HPH1F	ACGAGGTCGCCAACATCTTCTTCT
*AcOTAbZIP* terminator	AcOTAbZIP_2R	CACTCGCTCCTCCGTGATAT
	HPHPRO4	GCACCAAGCAGCAGATGATA
Hygromycin B	HMBF1	CTGTCGAGAAGTTTCTGATCG
	HMBR1	CTGATAGAGTTGGTCAAGACC
*AcOTAbZIP*	AcOTAbZIP_3F	CATCCATGCCCCAATTCGAG
	AcOTAbZIP_4R	TGCTTGAGGTCTAAGAGTTCCT
T-DNA copy numbers integrated into *A. carbonarius* Δ*AcOTAbZIP* strains genome		
*AcOTAbZIP*	AcOTAbZIP_CN_F	AATTGACAGCGAGGCGAATC
	AcOTAbZIP_CN_R	CCTGCAGCAACTCGATCAAA
Calmodulin	Cal_CN_F	CCTTACCATGATGGCTCG
	Cal_CN_R	TTCTCACCGATGGAGGTCAT
RT-PCR and RTqPCR (AC49 and Δ*AcOTAbZIP* strains)		
*AcOTAbZIP*	bZIPFor	TTTCCCTAGGATCTCTCCTA
	bZIPRev	TATTGGGGTCGGACAGGAAT
*AcOTApks*	pks4For	TCTGTATGAGCGCATCGCC
	pks4Rev	GCAGAAGGCCACTTTCCAG
*AcOTAnrps*	nrps6For	GATTCCGATGGAACTGCAAT
	nrps6Rev	CTGCCCCAGCATATCAATCT
*AcOTAP450*	P450For	GCCATACCTGACCGGGATCA
	P450Rev	GGGAAAATGGTCTCGTCGTG
*AcOTAhal*	halFor	AAAGAAGCCTACACCGACTT
	halRev	GAATTCGATGGATCCCGTGC
Ubiquitin	ubFor	CCGAAGGTCAACTTCACCAC
	ubRev	GGCATATTTGCGAGTCCATT

Bold: part of the primer useful for the treatment with the USER enzyme mix in the generation of 3′ single-stranded overhangs.

## Data Availability

Data is contained within the article or supplementary material.

## Supplementary Materials

The following are available online at https://www.mdpi.com/2072-6651/13/2/111/s1, Table S1: Location of the putative-OTA-gene cluster in the genome of the *Aspergillus* species and *Penicillium nordicum*. * position of OTA-gene cluster in the fungal genome (genome.jgi.doe.gov) identified based on homology with OTA putative gene cluster of *A. carbonarius*. Table S2: Features of BRLZ domains used in the Maximum Likelihood phylogenetic analysis. Table S3: Detail of the Transcription factor binding motif (TFBM) identified by MEME in the OTA-gene cluster upstream, downstream, and intergenic sequences. Table S4: TOMTOM analysis representing the homology of TFBM identified by MEME with those of *Saccharomyces cerevisiae*. * Name of transcription factor binding motif (TFBM) according to the JASPAR database.
