# Supplementary material for: Functional Role of Aspergillus carbonarius AcOTAbZIP Gene, a bZIP Transcription Factor within the OTA Gene Cluster
Source: Toxins (Basel). 2021 Feb 2;13(2):111. doi: 10.3390/toxins13020111 (PMC7913050; doi:10.3390/toxins13020111)
Supplement: Supplementary file 1 [file toxins-13-00111-s001.pdf]

Supplementary Materials: Functional Role of *Aspergillus carbonarius AcOTAbZIP* Gene, a bZIP Transcription Factor within the OTA Gene Cluster

Donato Gerin, Federica Garrapa, Ana-Rosa Ballester, Luis González-Candelas, Rita Milvia De Miccolis Angelini, Francesco Faretra and Stefania Pollastro

Table S1. Location of the putative-OTA-gene cluster in the genome of the *Aspergillus* species and *Penicillium nordicum*.

Species (Strain)	Putative OTA-Gene Cluster	
	Position*	Length (bp)
A. affinis (CBS 129190)	scaffold 263: 13,600–37,157	23,557
A. albertensis (IBT 14317)	scaffold 45: 173,601–196,151	22,550
A. alliaceus (CBS 536.65)	scaffold 38: 62,845–85,005	22,160
A. carbonarius (ITEM 5010)	scaffold 12: 943,452–965,219	21,767
A. cretensis (CBS 112802)	scaffold 236: 237–24,459	24,223
A. elegans (CBS 116.39)	scaffold 6: 221,286–244,294	23,008
A. flocculosus (CBS 112785)	scaffold 15: 210,525–234,022	23,497
A. muricatus (CBS 112808)	scaffold 98: 4,901–29,046	24,145
A. niger (*lacticoffeatus*) (CBS 101883)	scaffold 40: 220,217–241,951	21,734
A. niger (CBS 513.88)	supercontig 15: 1,855,355–1,877,016	21,661
A. niger (ATCC 13496)	scaffold 10: 59,997–82,127	22,130
A. ochraceus (fc-1)	Scaffold 8: 411,427–436,227	24,800
A. pulvericola (CBS 137327)	scaffold 10: 164,973–189,210	24,237
A. roseoglobulosus (CBS112800)	scaffold 81: 55,095–78,939	23,884
A. sclerotiicarbonarius (CBS 121057)	scaffold 107: 8,257–27,296	19,039
A. sclerotioniger (CBS 115572)	scaffold 10: 314,539–335,789	21,250
A. steynii (IBT 23096)	scaffold 3: 425,017–448,672	23,655
A. subramanianii (CBS 138230)	scaffold 42: 121,882–145,732	23,850
A. welwitschiae (CBS139.54b)	scaffold 25: 369,429–391,102	21,673
A. westerdijkiae (CBS 112803)	scaffold 73: 110,154–134,954	24,800
P. nordicum (DAOMC 185683)	scaffold 70: 4,687–26,262	21,575

*position of OTA-gene cluster in the fungal genome (genome.jgi.doe.gov) identified based on homology with OTA putative gene cluster of *A. carbonarius*.

Table S2. Features of BRLZ domains used in the Maximum Likelihood phylogenetic analysis.

Species (Strain)	Protein ID[a]	Sequence of BRLZ Domain[b]
		OTAbZIPs
A. affinis (CBS 129190)	267643	EVKQRRQKYHEKYKERNRLAAGKSRQKQVDLIALLE-AERSDEERRRRALEDEIQKIQKDLYAIKQELLH
A. albertensis (IBT 14317)	155937	KYHEKYKERNRLAAGRSRQKQVDLIELLEAERREEERRR-KALEEEIQRIQKELLAMKEELRH
A. alliaceus (CBS 536.65)	313480	KYHEKYKERNRLAAGRSRQKQVDLIELLEAERREEERRR-KALEEEIQRIQKELLAMKEELRH
A. carbonarius (ITEM 5010)	7821	KYHEKYKERNRVAAGKSRQKQVDLIELLQAEQREEERRR-KALERELSQIHKELLDLKQELQH
A. cretensis (CBS 112802)	260188	EVKQRRQKYHEKYKERNRLAAGKSRQKQVDLIALLE-AERRDEERRRRALEDEIQKIQKDLYAIKQELLH
A. elegans (CBS 116.39)	385076	KYHEKYKERNRLAAGKSRQKQVDLIALLE-

		AERRDEERRRRRALEDEIRQIQKELYAIKQELLH
A. flocculosus (CBS 112785)	228703	KYHEKYKERNRLAAGKSRQKQVDLIALLE-AERRDEERRRRALEDEIQKIQKDLYAIKQELLH
A. muricatus (CBS 112808)	167285	KYHEKYKERNRLAAGKSRQKQVDLIALLEAERRDEERRRQAL-EGEIQQIQKELYAIKQELHS
A. niger (ATCC 13496)	368844	KYHEKYKERNRLAAGRSRQKQADLINLLQAEQQEEERRRKALE-LEIANMQKELVDMKQELQH
A. niger (CBS 513.88)	167295	KYHEKYKERNRLAAGRSRQKQADLINLLQAEQQEEERRRKALE-LEIANMQKELVDMKQELQH
A. niger (lacticoffeatus) (CBS 101883)	438778	KYHEKYKERNRLAAGRSRQKQADLINLLQAEQQEEERRRKALE-LEIANMQKELVDMKQELQH
A. ochraceus (fc-1)	OTAR1[c]	KYHEKYKERNRLAAGKSRQKQVDLIALLE-AERRDEERRRRALEDEIRKIQKDLLAIKQELHS
A. pulvericola (CBS 137327)	431723	KYHEKYKERNRLAAGKSRQKQVDLIALLEAERRDEERRRRALE-HEIQKIQKDLYAMKQELLH
A. roseoglobulosus (CBS 112800)	176400	SEEVKQLRRKYHEKYKERNRLAAGKSRQKQVDLIALLE-AERRDEERRRRALEDEIQQIQRDLYAIKQELHN
A. sclerotiicarbonarius (CBS 121057)	328118	KYHEKYKERNRLAAGRSRQKQVDLIELLQAEQREEERRRQALEKEI-AQIQKELEDMKQELQH
A. sclerotioniger (CBS 115572)	593114	KYHEKYKERNRLAAGKSRQKQADLIELLQAEQREEERRRKALEKEI-AQMQKDLVDMKQELQH
A. steynii (IBT 23096)	473593	KYHEKYKERNRLAAGKSRQKQVDLIALLE-AERRDEERRRRALEDEIRQIQKELYAIKQELLH
A. subramanianii (CBS 138230)	274032	SDEVKQLRRKYHEKYKERNRLAASKSRQKQVDLIALLEAERG-DEERRRRALEDEIRQIQRDLHAIKQELHN
A. welwitschiae (CBS 139.54b)	172341	KYHEKYKERNRLAAGRSRQKQADLINLLQAEQQEEERRR-KALEQEIANMHKELVDMKQELYH
A. westerdijkiae (CBS 112803)	245987	KYHEKYKERNRLAAGKSRQKQVDLIALLE-AERRDEERRRRALEDEIRKIQKDLLAIKQELHS
P. nordicum (DAOMC 185683)	11413	KYHEKYKERNRLAAGKSRQKQVDLIALLEAERRDEERRRRVLEEEIQKIK-KDLFAIKQELHH
		A. carbonarius bZIPs
A. carbonarius (ITEM 5010)	132388	DSRERRKREHFLERNRVAANKCRK-KKKEHAKQLESRCEMVSRQNTLLESEVDHLKGEILNLKNELLR
	203634	TDEEKRKNFLERNRVAALKCRQRKKQWLANLQAKVELFTSENDALTAT-VTQLREEIVNLKTLLLA
	204486	LTSEPTSKRKAQNRAAQRAFRERKEKHLKDLETKVDELQAAS-DNANQENGLLRAQIERLQVELRE
	207072	DSADPAAMKRARNTEAARKSRARKLERQGEMERRIEELEKL-LEESQQREEYWRNVAENGS
	207738	KKLSSKERRQLRNKVSARAFRSRRKEYIGQLESEVAAKTNEA-HELRLQNRALFEENARLTDLARM
	208150	EKEQRRIERVLRNRAAAQTSRERKRLE-MEKLENEKIQMEQQNQFLLQRLSQMEAENNRLNQQIAQ
	208259	EEKESLT-PAQSKRKAQNRAAQRAFRERKERHVRELEEKVSNLENESTSLMADNERLKRELAKFTTENEI
	208905	ATDTPPTKRKAQNRAAQRAFRERRAARVNELEDQIKKIEDDHEIHVAT-FKDQISTLSLEVEQCRSEMAW
	209595	KRPLSTSKRAAQNRAAQRAFRQRKESYIRKLEEQVKEFEAT-

		NEALKQLQSENYQLREYIIN
	209790	ADRIKREKFLERNRVAASKCRHKKKLHTEM-LQHCHDDLSKKKTELSVLADKLRSELLALKNELLQ
	506085	IKELKQQKRLLRNRQAALDSRQRKKLHTEKLEEEKKQFTQVISDLEEEL-QNMRLREAELIREKGE

a: jgi.doe.gov. b: BRLZ domain obtained by using SMART (http://smart.embl-heidelberg.de/) and used for the Maximum Likelihood phylogenetic analysis. c: OTAR1 protein sequence obtained from its nucleotide sequence by using ExPaSy translation tool (http://expasy.org/tools/dna.html). The OTAR1 nucleotide sequence of *A. westerdijkiae* fc-1 was identified by BLASTn analysis on the assembled genome (www.ncbi.nlm.nih.gov/assembly/GCA_004849945.1) using the *A. westerdijkiae* CBS 112803 OTAbZIP sequence (ID: 245987, jgi.doe.gov) as query.

Table S3. Detail of the Transcription factor binding motif (TFBM) identified by MEME in the OTA-gene cluster upstream, downstream and intergenic sequences.

Fungal Species	Genomic Region*	Strand	Start	*p*-Value	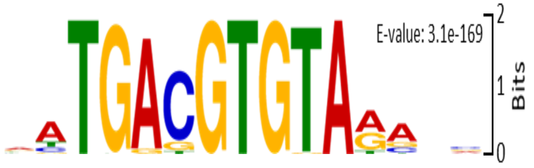
A. affinis	*OTApks/hp*	+	94	$6.93\times\times10^{-7}$	A T T G A C G T G T A G G A A
A. affinis	*OTApks/hp*	+	114	4.37×10^{-6}	C A T G A C G T G T A T T G C
A. affinis	*OTAnps/OTAp450*	+	323	6.59×10^{-8}	T A T G A C G T G T A A A T C
A. affinis	*OTAnps/OTAp450*	+	354	3.44×10^{-7}	A A T G A G G T G T A G A A C
A. affinis	*OTAp450/OTAbZIP*	−	714	2.51×10^{-9}	A A T G A C G T G T A A A A C
A. albertensis	*OTApks/hp*	−	66	7.53×10^{-9}	A A T G A C G T G T A G A T C
A. albertensis	*OTApks/hp*	−	86	6.03×10^{-6}	G C T G A C G T G G A A A T G
A. albertensis	*OTAnps/OTAhal*	−	154	3.33×10^{-8}	A A T G A C G T G T A A A G A
A. albertensis	*OTAhal/OTAp450*	+	9	1.76×10^{-6}	A C T G A C G T G T A G G A T
A. alliaceus	*OTApks/hp*	+	174	7.53×10^{-9}	A A T G A C G T G T A G A T C
A. alliaceus	*OTApks/hp*	+	154	6.03×10^{-6}	G C T G A C G T G G A A A T G
A. alliaceus	*OTAnps/OTAhal*	+	422	3.33×10^{-8}	A A T G A C G T G T A A A G A
A. alliaceus	*OTAhal/OTAp450*	−	1648	2.05×10^{-6}	A C T G A C G T G T A G G G T
A. alliaceus	*OTAhal/OTAp450*	−	1668	8.66×10^{-6}	A A T T A C G T G T A G A T T
A. alliaceus	*OTAp450/OTAbZIP*	−	706	1.60×10^{-7}	A A T G A C G T G T A A A A T
A. carbonarius	*OTApks/hp*	−	100	2.48×10^{-7}	G C T G A C G T G T A A A T C
A. carbonarius	*OTApks/hp*	−	79	8.99×10^{-7}	G T T G A C G T G T A G G G C
A. carbonarius	*OTAnps/OTAp450*	+	402	1.87×10^{-7}	A A T G A C G T G T A A C C C
A. carbonarius	*OTAp450/OTAbZIP*	+	110	1.72×10^{-8}	A A T G A C G T G T A A A A A
A. carbonarius	*OTAhal* (UTR-5')	−	408	1.38×10^{-6}	C T T G A C G T G T A A G C C

A. carbonarius	*OTAhal* (UTR-3')	+	407	5.47× 10^{-6}	A G T G A G G T G T A A G T G
A. carbonarius	*OTAhal* (UTR-5')	−	428	5.47× 10^{-6}	T G T G A C G T G T A G G A T
A. cretensis	*OTApks/hp*	−	67	6.93× 10^{-7}	A T T G A C G T G T A G G A A
A. cretensis	*OTApks/hp*	−	47	4.37× 10^{-6}	C A T G A C G T G T A T T G C
A. cretensis	*OTAnps/OTAp450*	−	134	3.44× 10^{-7}	T A T G A C G T G T A A C T C
A. cretensis	*OTAnps/OTAp450*	−	103	3.44× 10^{-7}	A A T G A G G T G T A G A A C
A. cretensis	*OTAp450/OTAbZIP*	+	85	2.51× 10^{-9}	A A T G A C G T G T A A A A C
A. cretensis	*OTAbZIP/OTAhal*	−	1720	2.19× 10^{-7}	G A T G A C G T G T A A G T C
A. elegans	*OTApks/OTAnps*	+	697	5.83× 10^{-7}	A T T G A C G T G T A G G A G
A. elegans	*OTApks/OTAnps*	+	717	1.91× 10^{-6}	C A T G A C G T G T A T C C C
A. elegans	*OTAnps/OTAp450*	+	421	5.18× 10^{-7}	T A T G A C G T G T A A C T A
A. elegans	*OTAnps/OTAp450*	+	452	5.83× 10^{-7}	G A T G A G G T G T A G A A C
A. elegans	*OTAp450/OTAbZIP*	−	921	1.38× 10^{-7}	T A T G A C G T G T A A A G A
A. elegans	*OTAbZIP/OTAhal*	+	171	7.53× 10^{-9}	A A T G A C G T G T A A A A G
A. elegans	*OTAbZIP/OTAhal*	+	734	6.93× 10^{-7}	A A T G A C G T G T A A G T T
A. flocculosus	*OTApks/hp*	−	211	1.13× 10^{-6}	T T T G A C G T G T A G G A G
A. flocculosus	*OTApks/hp*	−	191	4.37× 10^{-6}	C A T G A C G T G T A T T G C
A. flocculosus	*OTAnps/OTAp450*	−	167	4.57× 10^{-7}	A A T G A G G T G T A G A G C
A. flocculosus	*OTAnps/OTAp450*	−	198	5.83× 10^{-7}	T A T G A C G T G T A A C C G
A. flocculosus	*OTAp450/OTAbZIP*	+	119	1.38×10^{-7}	T A T G A C G T G T A A A G A
A. flocculosus	*OTAbZIP/OTAhal*	−	2295	1.16×10^{-7}	G A T G A C G T G T A A A C A
A. flocculosus	*OTAbZIP/OTAhal*	−	1901	1.91×10^{-6}	G G T G A C G T G T A A G A C
A. muricatus	*OTApks/hp*	−	135	2.19×10^{-7}	A T T G A C G T G T A G A T A
A. muricatus	*OTApks/hp*	−	115	2.36×10^{-6}	C A T G A C G T G T A T C C A
A. muricatus	*OTAnps/OTAp450*	−	173	4.57×10^{-7}	A A T G A G G T G T A G A G C
A. muricatus	*OTAnps/OTAp450*	−	204	1.91×10^{-6}	T A T G A C G T G T A A T T A
A. muricatus	*OTAp450/OTAbZIP*	+	121	1.87×10^{-7}	A A T G A C G T G T A G A T T
A. muricatus	*OTAbZIP/OTAhal*	−	1858	2.44×10^{-8}	A A T G A C G T G T A A A T A
A. niger (ATCC 13496)	*OTApks/hp*	−	144	2.19×10^{-7}	A T T G A C G T G T A A A T A

A. niger (ATCC 13496)	*OTApks/hp*	−	123	$3.44×10^{-7}$	G T T G A C G T G T A G A G G
A. niger (ATCC 13496)	*OTApks/hp*	+	107	$2.53×10^{-6}$	A C T G G C G T G T A G A T G
A. niger (ATCC 13496)	*OTAnps/OTAp450*	−	114	$5.18×10^{-7}$	T A T G A C G T G T A A C C C
A. niger (ATCC 13496)	*OTAnps/OTAp450*	−	94	$6.36×10^{-7}$	G A T G A G G T G T A G A A G
A. niger (ATCC 13496)	*OTAp450/OTAbZIP*	+	100	$3.33×10^{-8}$	G A T G A C G T G T A G A A G
A. niger (ATCC 13496)	*after /OTAhal*	−	1042	$4.37×10^{-6}$	C C T G A T G T G T A G A G C
A. niger (CBS 513.88)	*OTApks/OTAnps*	+	941	$2.19×10^{-7}$	A T T G A C G T G T A A A T A
A. niger (CBS 513.88)	*OTApks/OTAnps*	+	962	$3.44×10^{-7}$	G T T G A C G T G T A G A G G
A. niger (CBS 513.88)	*OTApks/OTAnps*	−	978	$2.53×10^{-6}$	A C T G G C G T G T A G A T G
A. niger (CBS 513.88)	*OTAnps/OTAp450*	+	401	$5.18×10^{-7}$	T A T G A C G T G T A A C C C
A. niger (CBS 513.88)	*OTAnps/OTAp450*	+	421	$6.36×10^{-7}$	G A T G A G G T G T A G A A G
A. niger (CBS 513.88)	*OTAp450/OTAbZIP*	−	836	$3.33×10^{-8}$	G A T G A C G T G T A G A A G
A. niger (CBS 513.88)	*OTAbZIP/OTAhal*	+	152	$2.19×10^{-7}$	A A T G A C G T G T A A G G A
A. niger (CBS 513.88)	*OTAbZIP/OTAhal*	+	173	$1.52×10^{-6}$	C C T G A C G T G T A A C C G
A. niger (CBS 513.88)	*after /OTAhal*	+	40	$4.37×10^{-6}$	C C T G A T G T G T A G A G C
A. niger (lacticoffeatus)	*OTApks/OTAnps*	+	1001	$2.19×10^{-7}$	A T T G A C G T G T A A A T A
A. niger (lacticoffeatus)	*OTApks/OTAnps*	+	1022	$3.44×10^{-7}$	G T T G A C G T G T A G A G G
A. niger (lacticoffeatus)	*OTApks/OTAnps*	−	1038	$2.53×10^{-6}$	A C T G G C G T G T A G A T G
A. niger (lacticoffeatus)	*OTAnps/OTAp450*	+	401	$5.18×10^{-7}$	T A T G A C G T G T A A C C C
A. niger (lacticoffeatus)	*OTAnps/OTAp450*	+	421	$6.36×10^{-7}$	G A T G A G G T G T A G A A G
A. niger (lacticoffeatus)	*OTAp450/OTAbZIP*	−	836	$3.33×10^{-8}$	G A T G A C G T G T A G A A G
A. niger (lacticoffeatus)	*OTAbZIP/OTAhal*	+	152	$2.19×10^{-7}$	A A T G A C G T G T A A G G A
A. niger (lacticoffeatus)	*OTAbZIP/OTAhal*	+	173	$1.52×10^{-6}$	C C T G A C G T G T A A C C G
A. niger (lacticoffeatus)	*after /OTAhal*	+	7	$4.37×10^{-6}$	C C T G A T G T G T A G A G C
A. pulvericola	*OTAnps/OTAp450*	−	168	$4.57×10^{-7}$	A A T G A G G T G T A G A G C
A. pulvericola	*OTAnps/OTAp450*	−	199	$5.83×10^{-7}$	T A T G A C G T G T A A C C G
A. pulvericola	*OTAp450/OTAbZIP*	+	28	$4.57×10^{-7}$	T A T G A C G T G T A A G A A
A. pulvericola	*OTAbZIP/OTAhal*	−	1474	$1.91×10^{-6}$	G G T G A C G T G T A A G A C
A. roseoglobulosus	*OTApks/hp*	−	61	$5.18×10^{-7}$	C A T G A C G T G T A T A A C
A. roseoglobulosus	*OTAnps/OTAp450*	−	8	$5.18×10^{-7}$	A A T G A G G T G T A G A A A
A. roseoglobulosus	*OTAnps/OTAp450*	−	40	$7.87×10^{-8}$	T A T G A C G T G T A A A T G

A. roseoglobulosus	*OTAp450/OTAbZIP*	+	55	4.03×10^{-7}	C A T G A C G T G T A A A A T
A. sclerotiicarbonarius	*OTApks/hp*	−	145	2.05×10^{-6}	A T T G A C G T G T A G G G T
A. sclerotiicarbonarius	*OTApks/hp*	+	128	7.46×10^{-6}	G C T G G C G T G T A T A T C
A. sclerotiicarbonarius	*OTApks/hp*	−	165	1.38×10^{-7}	A C T G A C G T G T A A A T C
A. sclerotiicarbonarius	*OTAnps/OTAp450*	−	131	1.16×10^{-7}	G A T G A C G T G T A A A C A
A. sclerotiicarbonarius	*OTAnps/OTAp450*	−	109	1.13×10^{-6}	A A T G A T G T G T A G A A G
A. sclerotiicarbonarius	*OTAbZIP/OTAhal*	−	475	1.13×10^{-6}	C C T G A C G T G T A A C T G
A. sclerotiicarbonarius	*OTAbZIP/OTAhal*	−	495	1.25×10^{-6}	T G T G A C G T G T A A A T G
A. sclerotioniger	*OTApks/hp*	+	235	6.36×10^{-7}	A T T G A C G T G T A G G G C
A. sclerotioniger	*OTApks/hp*	+	215	1.13×10^{-6}	T G T G A C G T G T A A A T C
A. sclerotioniger	*OTApks/hp*	−	251	8.25×10^{-6}	C T T G G C G T G T A T A T C
A. sclerotioniger	*OTAnps/OTAp450*	+	526	1.87×10^{-7}	A A T G A C G T G T A A C C C
A. sclerotioniger	*OTAnps/OTAp450*	+	557	1.91×10^{-6}	G A T G A T G T G T A A A A A
A. sclerotioniger	*OTAp450/OTAbZIP*	−	515	6.59×10^{-8}	G A T G A C G T G T A G A C C
A. sclerotioniger	*OTAbZIP/OTAhal*	+	159	1.76×10^{-6}	C T T G A C G T G T A G C C A
A. sclerotioniger	*OTAbZIP/OTAhal*	+	139	3.33×10^{-6}	T G T G A C G T G T A G A G T
A. steynii	*OTApks/hp*	+	164	5.83×10^{-7}	A T T G A C G T G T A G G A G
A. steynii	*OTApks/hp*	+	184	1.91×10^{-6}	C A T G A C G T G T A T C C C
A. steynii	*OTAnps/OTAp450*	+	539	5.18×10^{-7}	T A T G A C G T G T A A C T A
A. steynii	*OTAnps/OTAp450*	+	570	5.83×10^{-7}	G A T G A G G T G T A G A A C
A. steynii	*OTAp450/OTAbZIP*	−	922	6.59×10^{-8}	T A T G A C G T G T A A A A G
A. steynii	*OTAbZIP/OTAhal*	+	171	7.53×10^{-9}	A A T G A C G T G T A A A A G
A. steynii	*OTAbZIP/OTAhal*	+	734	1.38×10^{-7}	A A T G A C G T G T A A G T G
A. subramanianii	*OTApks/hp*	−	144	1.60×10^{-7}	A T T G A C G T G T A G A T G
A. subramanianii	*OTApks/hp*	−	124	3.56×10^{-6}	C A T G A C G T G T A T C A T
A. subramanianii	*OTAnps/OTAp450*	−	173	4.03×10^{-7}	A A T G A G G T G T A G A A G
A. subramanianii	*OTAnps/OTAp450*	−	204	5.18×10^{-7}	T A T G A C G T G T A A C T A
A. subramanianii	*OTAp450/OTAbZIP*	+	95	4.03×10^{-7}	C A T G A C G T G T A A A A T
A. subramanianii	*OTAbZIP/OTAhal*	−	2118	5.28×10^{-8}	G A T G A C G T G T A A A A A
A. welwitschiae	*OTApks/OTAnps*	+	949	2.19×10^{-7}	A T T G A C G T G T A A A T A
A. welwitschiae	*OTApks/OTAnps*	+	970	3.44×10^{-7}	G T T G A C G T G T A G A G G

A. welwitschiae	*OTApks/OTAnps*	−	986	2.53×10^{-6}	A C T G G C G T G T A G A T G
A. welwitschiae	*OTAnps/OTAp450*	+	381	5.18×10^{-7}	T A T G A C G T G T A A C C C
A. welwitschiae	*OTAnps/OTAp450*	+	401	6.36×10^{-7}	G A T G A G G T G T A G A A G
A. welwitschiae	*OTAp450/OTAbZIP*	−	836	3.33×10^{-8}	G A T G A C G T G T A G A A G
A. welwitschiae	*OTAbZIP/OTAhal*	+	152	2.19×10^{-7}	A A T G A C G T G T A A G G A
A. welwitschiae	*OTAbZIP/OTAhal*	+	173	1.76×10^{-6}	C C T G A C G T G T A A C C A
A. welwitschiae	*after /OTAhal*	+	40	4.37×10^{-6}	C C T G A T G T G T A G A G C
A. westerdijkiae	*OTApks/hp*	−	164	3.44×10^{-7}	A T T G A C G T G T A G A C A
A. westerdijkiae	*OTApks/hp*	−	144	1.52×10^{-6}	C A T G A C G T G T A T C A G
A. westerdijkiae	*OTAnps/OTAp450*	−	16	5.18×10^{-7}	T A T G A C G T G T A A C T A
A. westerdijkiae	*OTAp450/OTAbZIP*	+	110	1.16×10^{-7}	T A T G A C G T G T A A A T A
A. westerdijkiae	*OTAbZIP/OTAhal*	−	2726	7.53×10^{-9}	A A T G A C G T G T A A A A G
A. westerdijkiae	*OTAbZIP/OTAhal*	−	2592	8.99×10^{-7}	A A T G A C G T G T A A T A A
P. nordicum	*OTApks/hp*	+	174	3.44×10^{-7}	A T T G A C G T G T A G A C A
P. nordicum	*OTApks/hp*	+	194	6.35×10^{-6}	A A T G A C G T G T A T T G T
P. nordicum	*OTAp450/OTAbZIP*	−	669	1.16×10^{-7}	C A T G A C G T G T A A A G A
P. nordicum	*OTAbZIP/OTAhal*	+	548	2.19×10^{-7}	G A T G A C G T G T A A G T C
P.. nordicum	*OTAbZIP/OTAhal*	+	159	5.28×10^{-8}	A A T G A C G T G T A A A C A

*For each fungal strain, the genomic regions used for the analysis were the intergenic regions between the reported genes, or corresponded to the untranslated regions (UTR) of the specified genes. *OTApks*: polyketide synthese; *OTAnps*: nonribosomal peptide synthase; *OTAp450*: cytochrome P450 monooxygenase; *OTAbZIP*: bZIP transcription factor; *OTAhal*: halogenase; *hp* means the hypothetical protein between the polyketide synthese (*OTApks*) and the nonribosomal peptide synthase (*OTAnps*).

Table S4. TOMTOM analysis representing the homology of TFBM identified by MEME with those of *Saccaromyces cerevisiae*.

Name*	Transcription Factor Class	*p*-Value	*E*-Value	*q*-Value
MA0351.1 (DOT6)	Tryptophan cluster factors	4.36×10^{-4}	7.68×10^{-2}	1.51×10^{-1}
MA0409.1 (TYE7)	Basic helix-loop-helix factors (bHLH)	2.21×10^{-3}	3.89×10^{-1}	3.54×10^{-1}
MA0318.1 (HMRA2)	TALE-type homeo domain factors	3.07×10^{-3}	5.41×10^{-1}	3.54×10^{-1}
MA0328.2 (MATALPHA2)	TALE-type homeo domain factors	4.12×10^{-3}	7.25×10^{-1}	3.55×10^{-1}
MA0421.1 (NSI1)	Tryptophan cluster factors	5.32×10^{-3}	9.37×10^{-1}	3.68×10^{-1}
MA0281.1 (CBF1)	Basic helix-loop-helix factors (bHLH)	8.12×10^{-3}	1.43	4.27×10^{-1}
MA0329.1 (MBP1)	APSES-type DNA-binding domain	9.59×10^{-3}	1.69	4.27×10^{-1}
MA0286.1 (CST6)	Basic leucine zipper factors (bZIP)	1.04×10^{-2}	1.83	4.27×10^{-1}
MA0416.1 (YAP3)	Basic leucine zipper factors (bZIP)	1.25×10^{-2}	2.20	4.27×10^{-1}
MA0350.1 (TOD6)	Tryptophan cluster factors	1.42×10^{-2}	2.50	4.27×10^{-1}
MA0310.1 (HAC1)	Basic leucine zipper factors (bZIP)	1.45×10^{-2}	2.55	4.27×10^{-1}

*Name of transcription factor binding motif (TFBM) according to the JASPAR database.
